# Courtesy Among Dentists at Professional Meetings

**Published:** 1887-02

**Authors:** J. N. Farrar

**Affiliations:** New York City


### COURTESY AMONG DENTISTS AT PROFESSIONAL MEETINGS.

An Annual Oration Delivered Before the American Academy of Dental Science, Boston, Mass., Nov. 10th, 1886.

BY J. N. FARRAR, M. D., D. D. S., NEW YORK CITY.

*Mr. President and Gentlemen:*

It is with misgivings that I attempt to speak upon a subject from which people shrink, because I do not feel able to put it as forcibly as it deserves. But I esteem it a duty to ally myself with others in the battle against error, and in the support of principles necessary to the advancement of the profession, notwithstanding it may be unpleasant.

I trust that my remarks will not be taken in any other than a kindly spirit, as they are intended only for general good, through the influence of my hearers, wherever they may go. I say this because I do not wish it thought that references are made to this Society, concerning which I have never heard aught but good.

Although I may offer nothing new, I hope, by pointing out some practices, to place the matter in such a light that the motives behind them may be recognized whenever present, with the view of correcting evils that would make the profession blush with shame if they were publicly set forth by some prominent writer of fiction. I refer to the objectionable methods that have been too frequently practiced tending to hurt personal feelings, or injure personal standing. It is not my intention to dwell upon the science as a science, or as regards professional courtesy in and through business, or the damaging and paralyzing influence of political "wire pulling" at elections, but my remarks will have reference more especially to graver conduct; the crippling and assassination of genius, which has been practiced for years in the meetings of societies: not that other professions are exempt from this blight, but that ours is the one with which we have principally to deal.

Sometimes this is conducted in an open warlike manner, at others more in the style of intrigue under the guise of "love for truth;" not within the limit prescribed by the spirit of Mosaic doctrine, "a tooth for a tooth," but too often in the spirit borne by the hawk toward the innocent dove ; unlike the good teacher, who seeks to draw out the latent power of the listener's judgment by kindly criticism upon the subject, the critic too often indirectly aims his shafts at the speaker personally, with intent to injure his popularity.

It may be thought that the only improvement dental societies need in their meetings is to talk less and say more ; but, however true the saying,

> "They never taste who always drink,
> They always talk who never think,"

it is not enough for the cure of this evil.

The influence of courtesy is wider and reaches deeper into business, as well as social life, than is commonly supposed. He who recognizes favor merely on one side of a business transaction, fails to understand not only what constitutes honor, but the commonest principles underlying society. A merchant who does not know that his profits come through a form of courtesy of the purchaser, cannot understand why he should show courtesy to an honest man who has been prompt pay for years, by not crowding him in the time of distress. One who says, "I gave him his money's worth, therefore he has no claims upon me," should not feel hard toward an old customer if he should leave him and patronize another who has more humanity in his nature. Just so is it in professional life. He who shows no courtesy by making distinction between the conditions of patients, and who plays "the spider and the fly," and, taking advantage of their confidence, presents exorbitant bills out of all proportion to the value of service rendered, ought not to be surprised if, in the days of his gray hairs, he finds himself in want of funds, friends and business. So he who has no higher aim in discussion on scientific subjects than personal aggrandizement at the expense of others, not only is a " millstone that clogs the wheels of progress," but he grinds himself out of recognizance.

Unlike the people who lived in the days of Homer, when dark ignorance was the rule, most people are now able to detect the motive. While in olden times the masses, attracted by a few brilliant intellects, like meteoric lights, were led by them, the difference between the leaders and the led is not now so great. Instead of one or two bright stars here and there, there are now so many that this world seems more like a torchlight procession marching in grand array. A few tyrants still exist; but their thrones are dissolving away by the light of truth thrown upon them through the efforts of sincere and modest scientists, the class most loyal to the interests of the profession, and who would often do more if encouraged.

Born and reared not far from this city, in a town where public debates were common, and were conducted by rules of strict courtesy; where it appeared to be the fashion and desire to try to outdo one another in politeness, and to take no notice of those who dared show ill-feeling; where there seemed to be an understood though unwritten law that, to speak unkindly of others, whether in their presence or during their absence, was to dig one's own grave—was it strange that I should have been amazed when I went forth into the world and saw these principles violated so frequently as to cause such fear in the more timid minds, that they prefer to remain silent rather than to place themselves in the current of personal abuse of those who seem to feel that the strength of their citadel depends upon destroying that of others? Of course, since people differ in their dispositions, allowance should be made; but for the violation of the commonest rules of courtesy, there can be no reasonable excuse.

There are those who are gentlemen under all circumstances, wherever found; who do not think that, because others may conduct themselves improperly, it is a reason why they should; there are those who appear like gentlemen toward everybody in society at large, who carry none of it into meetings to show toward their brethren in discussion;—everything nice to the outer world, and even toward members of the profession individually met, but who forget that it is equally good policy, if for no higher reason, to show the same disposition in society meetings.

There are those who quarrel for the love of it, and who like to meet their equals, and there are those who delight in finding a timid person to climb upon, no matter how talented he may be. Such pugilistic spirits do not always appear to know when they are defeated in argument. They love debate, but cannot set themselves a-going without first throwing a club; like the boy who slings a stone at a cow to create an impression in his favor, and then rushes on.

These who delight in this appearance of altitude, and think it necessary to first silence those who presume to speak upon subjects on which they have thought themselves authority, are often paradoxical in their nature. I once knew a person of this kind, who, after shamefully abusing an essayist by belittling him, subsequently found his equal in the essayist, and being severely punished, in a second speech made eloquent remarks upon the foolishness of indulging in personalities in scientific meetings.

If they who fight openly are reprehensible, what shall we say of those who seek to destroy in a more stealthy manner; who, under cover of suavity and apparent politeness, carry the insinuating dagger?

Some work simply to destroy, without disposition to rebuild; others seek first to lower the structure of the opponent to a level with the ground, and then to rebuild for themselves. But to build a superior structure alongside that of another, so that the difference can be seen, is proof of superior ability, much more persuasive in its influence, and its fairness commands the respect of everybody, even of those who are not convinced. There is no such powerful means of progression towards personal elevation, as through a spirit of generosity and courtesy, shown in respect for the opinions of others. He who to himself would have courtesy shown, should first himself show it to others.

Courtesy is of two kinds; natural, and artificial. When courtesy is the expression of generous refinement, it may be said to be the perfection of gracefulness. But courtesy artificially attained is better than swinishness, for the same reason that deformity is less objectionable hidden than if exposed. Exposed mental deformity is even more objectionable than exposed physical deformity.

It does not follow that to express one's adverse views with effect, it must be done in a pugilistic manner. Politeness is a bond of good fellowship, and begets friends; while selfishness embitters the social atmosphere, and freezes the tender impulses. There is no spectacle so grand and noble as two opposing debaters facing each other as gentlemen, vying in politeness as if endeavoring to outdo each other in courtesy, though not descending to flattery. Adverse criticism, even if a little harsh, is more acceptable to a sensible opponent than treacle. I have known great undertakings, and elaborate preparation for scientific discussion made total failure by a few garrulous, quarrelsome, omnipresent spirits who consumed the time on unimportant things; such as parliamentary trifles: consuming time that might have been used to far greater advantage in the scientific discussion, for which the meeting was called, causing disturbance of mental equipoise, as shown in loss of interest and bitterness of feeling.

Understand me, I do not advocate ignorance of parliamentary rules, but to make the scientific object of the occasion subordinate to trifling and unimportant technicalities, is not conducive to the growth of scientific knowledge, nor is it satisfactory to the majority of the members.

Some might say, to guard against this drawback more of the better quality of material is necessary. That, to strike at the root of this evil, greater discrimination must be used in the selection of pupils to the profession, which implies more natural refinement and higher scholarship; a quality of men who would set their heel upon such conduct. Undoubtedly could the Utopian view be carried out, it would go far to remedy the evil; but our present object is to doctor ourselves, and then, having healthier and better blood, to try to infuse it into others.

Probably there is no better way of preventing this descent to personalities, than by the exercise of determination by the President; and I doubt if there is a society in this broad land that would not, as a whole, stand by him when he exercises it. Bullies make poor soldiers, and cower before legal power.

Conducted in accordance with these principles, a society will grow, socially and scientifically; but, in the proportion that the meetings fall below this standard, in that ratio will the righteous indignation of popular opinion sooner or later express its disapproval to such an extent that genius will absent itself, and the society crumble into decay through apathy.

There are other methods of committing this crime, as by boycotting the speaker by remaining away from the meeting, or being present with the sole view to destroy him through his subject by drawing his thoughts away from his theme; asking questions not relevant. Boycotting, however, is so obnoxious to the American that little need be said further than that this spirit, though not so rife as in former years, is not entirely extinct.

Probably the most cowardly way of all is the indulging in slurring personalities in public meetings against an absent party, who cannot defend himself. One of the most peculiar ways of making an attack upon an absent person, whose name cannot relevantly be dragged into the discussion of the meeting, is to have an understanding with some member of the society to call upon the enemy to express his views upon the subject in which the absent party is especially interested.

To assume superior wisdom, and to endeavor to overawe these pretenders presuming upon the ignorance of the audience, sometimes make bold statements on abstruse and uncommon phases of subjects,—assertions, the truth or falsity of which would require a year of hard experimental study to determine—this is a trick that I have known to be practiced more than once with wonderful success. Sometimes, however, a David rises up, and, with the fruits of patient toil, calmly slays the misleading hypotheses of such would-be Goliaths.

Having confined my previous remarks to the conduct of person to person, we now may come to the acts of members taken collectively, which the following incident may illustrate. In speaking with a prominent member of our profession regarding a certain measure endorsed by his society, he was asked how they could commit so unjust an act. His reply was this: "Probably there was not a member present at that meeting, who, if he had acted from his own heart, would have voted for the measure; but you know that sometimes men collectively dare to do things that individually they would be ashamed of."

To criticise the views of an essayist in an offensive manner, casting aspersions upon his mental ability, under cover of the pretense that the society will be held responsible for the influence his views may have upon the profession at large, if made public through the press, is probably one of the weakest excuses for displaying prejudice. The public generally gives credit to people for what they are worth, and does not place much stress upon the whereabouts of the speech. If erroneous views are set forth in societies that publish their proceedings, would it not be as well to leave that matter to the discretion of the editor and his basket, rather than to make rancorous speeches that injure the reputation of the society more than the essayist? It is bad enough to fight on one's own account, but for a member to place a society in the light of Balaam's friend, to ride to battle on, is not conducive to the growth or the popularity of the society, or to the advancement of the profession.

Not long ago a member of high standing, a man who is in every respect a gentleman, one whom you all know, after having read a very scholarly paper, was attacked in a bigoted manner on a point not at all relevant to the subject; and, in the "star-chamber method," was ill-treated in a manner not at all creditable to any society. This essayist afterwards said to me that he was always very glad to be the object of honest, intelligent criticism; but when criticism comes in such a shape to an invited guest he could not forget it, nor would he stoop to strike back in a spirit of wrath. This is not an exceptional case. I have known many such, and have lived long enough to see decay set into every society that tolerated it.

When a man is invited to speak before a society, he naturally expects to be treated in a gentlemanly manner, even though his ideas may be at variance with those of some, or even all, of its members. The value of such occasions often depends upon the remarks subsequently made by others in reply to the speaker; but courtesy demands that respect should be shown, not only in the criticisms, but also by absence of low grumblings, loud whisperings, or any other means of making a noise with intention to disturb the speaker, or to attract from him the attention of the audience. Silence and close attention are absolutely necessary to secure the best efforts of some speakers; especially if they know that, among the audience, there are those who do not sympathize with him. There speaks in New York every Sunday, one of the most eloquent orators of the nineteenth century, who is so easily thrown from his equipoise that he requires the outer door to be locked when he commences, that he may not be disturbed by stragglers.

Criticism is the life of a scientific meeting; but if I may be allowed to quote from myself:

> Virtues, by proper use,
> May become vices by abuse.

Personal abuse is not criticism, nor is faultfinding criticism; but to differ from one's ideas upon the subject and to show why, when done in a kindly and candid manner, is agreeable to an audience, and not objectionable to any right-minded speaker. By right-minded I mean more than having knowledge of the fact that, an excessive flatterer is a mercenary person, and one who cannot be relied upon in times of adversity, nor when character is wantonly attacked behind the back, but one whose love for truth and desire for the advancement of knowledge is closer at heart than some pet hypothesis or personal aggrandizement. The former comes from a spirit of generosity; the latter, jealousy. One is the open helping hand; the other, a clenched fist, disputing the right to step on ground it considers its own.

Brotherly love, shown in the courteous examination of a subject, stimulates freedom of intercourse, cultivates humanity of feeling and keeps down the pugnacious element. A method that builds up the truth with evidence, so that it looms above all surrounding error, instead of the old feudal method of attaining superiority through the destruction of others. Compeers make compeers by kindly feelings, through mutual support; while abuse kills the growth of the abuser by isolation, as much as if he were upon an uninhabited island, unknown, living to no purpose superior to that of the vegetable. No man can become great of himself alone, and without the sympathy of his fellows.

Admitting that all have their friends, and everybody of any account his enemies, and that to be a gentleman under all circumstances is the privilege of all; let us with a spirit of gentleness toward our brethren of different opinions, our actions showing that the expression of different views stimulates thought and a disposition to learn, let us, shoulder to shoulder, move on with solid front, with higher aims to higher ground, that we may command the world's greater respect.

